# A Key Management Scheme Based on Pairing-Free Identity Based Digital Signature Algorithm for Heterogeneous Wireless Sensor Networks

**DOI:** 10.3390/s20061543

**Published:** 2020-03-11

**Authors:** Erdong Yuan, Liejun Wang, Shuli Cheng, Naixiang Ao, Qingrui Guo

**Affiliations:** 1 School of Information Science and Engineering, Xinjiang University, Urumqi 830046, China; yuanerdong163@163.com (E.Y.); cslxjuedu@126.com (S.C.); 2 Xinjiang Lianhai INA-INT Information Technology Ltd., Urumqi 830000, China; aonaixiang163@163.com; 3 State Grid Xinjiang Electric Power Research Institute, Urumqi 830000, China; gur1008@163.com

**Keywords:** key management, security, relay node, PF-IBS, heterogeneous wireless sensor networks

## Abstract

The secure transmission of data within a network has received great attention. As the core of the security management mechanism, the key management scheme design needs further research. In view of the safety and energy consumption problems in recent papers, we propose a key management scheme based on the pairing-free identity based digital signature (PF-IBS) algorithm for heterogeneous wireless sensor networks (HWSNs). Our scheme uses the PF-IBS algorithm to complete message authentication, which is safer and more energy efficient than some recent schemes. Moreover, we use the base station (BS) as the processing center for the huge data in the network, thereby saving network energy consumption and improving the network life cycle. Finally, we indirectly prevent the attacker from capturing relay nodes that upload data between clusters in the network (some cluster head nodes cannot communicate directly). Through performance evaluation, the scheme we proposed reasonably sacrifices part of the storage space in exchange for entire network security while saving energy consumption.

## 1. Introduction

Wireless sensor networks (WSNs) can be applied in military, traffic management, smart healthcare, and smart homes, etc. [1]. For example, in terms of medical health [2], it has brought great convenience to our society. However, in the application process, the secure transmission of data is essential. If there is no secure data transmission in the network, it is easy for an attacker to obtain sensitive data in the network, or even forge some data to attack the network, which will lead to the confidentiality and integrity of the data collected by the network to be questioned. Key management can provide secure communication keys for information transfer between network nodes to ensure message confidentiality, integrity, and authentication, etc. 

Sensor network nodes are limited in terms of storage space, computing power, and energy reserves. As the symmetric encryption algorithm encryption and decryption process consumes less energy, this algorithm is often used for secure data transmission on the network, such as [3]. However, symmetric encryption algorithms cannot complete digital signature authentication. Traditional public key encryption algorithms (say RSA (Ron Rivest, Adi Shamirh and Len Adleman)) can only guarantee security when the key length (at least 1024 bits) is long enough. The increase in key length brings computational complexity. Considering the problems of large storage space, high computational complexity, and high energy consumption, traditional public key encryption algorithms were not considered suitable for sensor network nodes. However, through the improvement of the elliptic curve cryptography (ECC) algorithm and performing actual tests on the sensor nodes (such as [4,5]), it is proved that ECC can be applied to resource-constrained WSNs. Another issue to consider is that traditional public key infrastructure (PKI) issues a public key certificate for each node to help them verify the authenticity of the other party’s public key. However, the generation, storage, publishing, and verification of certificates also consume a lot of resources on the network. It is commendable that the emerging identity-based cryptography system solves this problem [6].

In this study, we learned that the heterogeneity of sensor nodes and sensor networks objectively exists. The internal node type in a homogeneous sensor network is single, and the network model often differs greatly from the actual application [7]. At the same time, the limitations of the homogeneous sensor network itself also limit the application of public key cryptosystems, hierarchical network management models, and other technologies in sensor networks. Therefore, in order to better study key management in sensor networks, the addition of heterogeneity has been favored by some researchers, such as [8,9,10]. We propose a key management scheme for heterogeneous wireless sensor networks (HWSNs). Our scheme makes some contributions:

1. Our scheme uses the pairing-free identity based digital signature (PF-IBS) algorithm to complete identity authentication. This algorithm not only ensures the security of the key establishment process, but also saves energy.
2. We adopted a new network model. The energy consumption of generating the network routing structure is borne by the base station (BS), which saves a lot of computing costs for the internal network nodes.
3. We protect the location privacy information of nodes in the network to prevent attackers from discovering and attacking relay nodes in the network.

The rest of the paper is organized as follows: Section 2 reviews some guiding key management schemes. Section 3 explains the proposed key management scheme for HWSNs. Section 4 evaluates the performance of the proposed scheme. Section 5 summarizes our paper.

## 2. Related Works

Du et al. [11] designed a distributed key management scheme combining a routing structure and ECC based on the scheme [12]. This scheme proposes the concept of a communication neighbor (c-neighbor), that is, the node only needs to establish the communication key with the c-neighbor node instead of establishing the communication key with all neighbor nodes. However, in order to obtain a c-neighbor relationship, the low-performance sensor (L-sensor) must upload its own location information to the high-performance sensor (H-sensor). The H-sensor generates an intra-cluster routing structure based on the collected location information of the L-sensor, and then distributes neighbor information for each L-sensor in the cluster. The advantage of this scheme is that the key establishment process combined with the routing structure can omit some unnecessary communication link establishment, thereby saving the computational cost and communication cost of the network; however, the disadvantage is that the communication load of the H-sensor is too large and takes up a lot of storage space. At the same time, the node lacks message authentication during the key establishment process.

Boujelben et al. [13] proposed an identity based key management scheme for heterogeneous sensor networks. This scheme assumes that each node knows the identity identifiers of all neighboring nodes in advance. Bilinear pairing based on identity cryptography (IBC) is used to assist in establishing the session key. For two nodes (say $N_{i}$ and $N_{j})$ that want to establish a session key $K_{ij}$, each node first uses its own private key and the other’s public key and combines the properties of bilinear mapping to generate a common key $V_{ij}$. Then, they use $V_{ij}$ to encrypt the message $(r_{i}P$ or $r_{j}P$) sent to the other party to generate a message authentication code, and send the message authentication code and message to the other party. Finally, when the message sent by the other party is verified, they use the Diffie–Hellman key exchange algorithm [14] to generate $K_{ij}.$ The advantage of this scheme is better security and less key storage occupancy. The disadvantage is that the key establishment process only has message authentication and no identity authentication, and the key establishment process consumes more energy.

Wang et al. [15] proposed a distributed key management scheme that makes communication links more secure. In this scheme, Wang et al. have taken some improvement measures to address the two issues in the scheme [11]. Wang et al. used an energy-aware routing protocol to improve the first problem. For the second problem, the identity-based encryption (IBE) algorithm is used for message authentication. The advantage of the scheme is that it achieves better security through message authentication during the key establishment process. At the same time, the energy-aware routing algorithm [16] is used to save network energy consumption. The disadvantage of the scheme is that using the IBE algorithm to complete message authentication will consume a lot of energy. At the same time, the message authentication process cannot defend against replay attacks.

Harbi et al. [17] proposed a key management scheme that can ensure network data transmission security by enhancing authentication during key establishment. The scheme found that the Inter-Cluster Multiple Key Distribution Scheme for Wireless Sensor Network (ICMDS) [18] has the problem where it cannot implement the calculation of the session key. After reviewing the design process of the ICMDS scheme, Habib et al. found that the master private key needed to establish the key was lacking. In response to the problem of the ICMDS scheme, Habib et al. designed a new key management scheme based on the identity-based encryption (IBE) algorithm. The advantage of this scheme is that it can resist multiple types of attacks. However, the disadvantage of this scheme is that once the master private key is known by the attacker, all session keys established within the network will be exposed. In addition, completing identity authentication will consume a lot of energy.

Through the analysis of the above schemes, we know that ensuring the security of the designed key management scheme has always been the focus of attention. However, the security problems of key management schemes still exist. Even though security can be guaranteed in some ways, it comes at the expense of energy consumption. At the same time, we learn that identity-based security mechanisms have also received great attention from researchers.

## 3. Proposed Key Management Scheme for HWSNs

### 3.1. PF-IBS Algorithm

We optimize the PF-IBS algorithm proposed by Sharma et al. [19] The public key of each node in our optimized PF-IBS algorithm is related to $ID_{i}$, which is more conducive to identity authentication. Its security depends on the intractability of the discrete logarithm on the elliptic curve. Its implementation requires the following four steps:

**Setup:**  PKG selects a safe elliptic curve $E/F_{p}$ over the finite field $F_{p}$: $y^{2}=x^{3}+\mathrm{ax}+b$, where a, b $\in F_{p}$ and $\Delta =4a^{3}+27b^{2}\neq 0$. $E/F_{p}$ satisfying $\Delta =4a^{3}+27b^{2}\neq 0$ is a non-singular hyperelliptic curve, which is suitable for cryptographic applications. $E\left(F_{p}\right)$ consists of points on an elliptic curve and points of infinity and constitutes a group. $P\in E\left(F_{p}\right)$ as the generator of G.
  (1) PKG selects $s\in Z_{q\;}^{*}$ as the master private key and obtains the master public key $P_{pub}=sP$.
  (2) PKG selects two hash functions: $H_{1}:{\left\{0,1\right\}}^{l_{ID_{i}}}\times G\to Z_{q}^{*}{\;\mathrm{and}\;H}_{2}:{\left\{0,1\right\}}^{l_{ID_{i}}}\times G\times {\left\{0,1\right\}}^{l_{m}}\to Z_{q}^{*}$, where $l_{ID_{i}}$ represents the length of $ID_{i}$ and $l_{m}$ represents the length of the message m.
  (3) PKG outputs public system parameter $\pi =\left\{E/F_{p},q,G,P,P_{pub},H_{1},H_{2}\right\}$.
**Extract:**  PKG inputs each node identity $ID_{i}\in {\left\{0,1\right\}}^{*},\pi$, and s.
  (1) PKG selects $r_{ID_{i}}\in Z_{q}^{*}$, and calculates RIDi=rIDi·P and cIDi=H1(IDi||RIDi), where $c_{ID_{i}}$ is the public key of each node.
  (2) PKG calculates $d_{ID_{i}}=r_{ID_{i}}+c_{ID_{i}}s\;mod\;q$, where $\left(R_{ID_{i}},d_{ID_{i}}\right)$ is the private key of the node.
  (3) PKG preloads $\left(R_{ID_{i}},d_{ID_{i}}\right)$ and π correspondingly into each node.
**Sign:**  Taking nodes u and v as an example, u signs the message $m_{u}$ with its private key $\left(R_{u},d_{u}\right)$ and sends $ID_{u}$, $m_{u}$, and the signed message $\sigma_{u}$ to v.
  (1) u chooses $a\;\mathrm{random}\;\mathrm{number}\;\varphi_{u}\in Z_{q}^{*}$, calculates $E_{u}=\varphi_{u}\cdot P$ first, and then calculates $h_{u}=H_{2}\left(m_{u},ID_{u},R_{u}\right)$ and $Z_{u}=\varphi_{u}+h_{u}\cdot d_{u}\;mod\;q$.
  (2) u generates $\sigma_{u}=\left(E_{u},R_{u},Z_{u}\right)$.
**Verify:**  v uses the received $ID_{u}$ to verify $\sigma_{u}$ sent by u.
  (1) v calculates $c_{u}=H_{1}(ID_{u}||R_{u})\;\mathrm{and}\;h_{u}=H_{2}\left(m_{u},\;ID_{u},R_{u}\right)$.
  (2) v determines whether the left and right sides of $Z_{u}\cdot P=E_{u}+h_{u}\cdot \left(R_{u}+c_{u}P_{pub}\right)$ are equal. If two sides are equal, $m_{u}$ and identity authentication pass, and vice versa.

### 3.2. Construction of Network Model

#### 3.2.1. Network Assumptions

Our network model includes a powerful BS, a few high-performance sensors (H-sensors), and many low-performance sensors (L-sensors). According to the needs of the network architecture, each H-sensor in the network will be used as the cluster head (CH). We have listed some assumptions about the network model:

(1) The L-sensor does not have tamper-resistant hardware. Once the opponent captures the L-sensor, all the important information stored in the L-sensor can be obtained. Due to cost issues, the L-sensors’ computing power, storage space, and energy are greatly limited.
(2) The H-sensor plays a key role in the transmission of sensor data within the cluster. The important information they store may affect the security of the L-sensor communication link within the cluster. Therefore, the H-sensor has a tamper-proof facility to better enhance the security of the network.
(3) BS has a high computing power, wide communication range, and enough storage space and energy.
(4) Each L-sensor and each H-sensor in the network has a unique identifier ${\mathrm{ID}}_{L_{i}}$ and ${\mathrm{ID}}_{H_{i}}$, respectively. The BS has its identifier ${\mathrm{ID}}_{BS}$. The identifiers of all L-sensors are denoted by ${\mathrm{ID}}_{L_{s}}$. The identifiers of all H-sensors are denoted by ${\mathrm{ID}}_{H_{s}}$. The identifiers of some L-sensors are represented by IDs.
(5) The BS is well protected and trusted.

#### 3.2.2. Network Communication Mode

Our network model draws on three communication modes used in LEAP+ [20]:

- Unicast (the process by which a particular node sends a message to a single node).
- Local broadcast (the process by which a particular node sends a message to all neighbor nodes within its communication range).
- Global broadcast (the process by which a particular node sends a message to all nodes in the network).

#### 3.2.3. Data Preloading of Network Nodes 

Based on the above assumptions that the BS is trusted and protected, we use the BS to act as a PKG role. The BS uses π and s in combination with ${\mathrm{ID}}_{L_{i}}$ to generate each L-sensor‘s identity-based public key $c_{L_{i}}$ and private key $\left(R_{L_{i}},d_{L_{i}}\right)$. The same operation process for the identity identifiers ${\mathrm{ID}}_{H_{i}}$ of each H-sensor and ${\mathrm{ID}}_{BS}$ of the BS can generate $c_{H_{i}}$, $\left(R_{H_{i}},d_{H_{i}}\right)$, and $c_{BS}$, $\left(R_{BS},d_{BS}\right)$ for them. The asymmetric pair keys of all L-sensors are denoted by $c_{L_{s}}$ and $\left(R_{L_{s}},d_{L_{s}}\right)$, respectively. Similar expression methods, $c_{H_{s}}$ and $\left(R_{H_{s}},d_{H_{s}}\right)$, will be used to represent the public and private keys of all H-sensors. Each L-sensor is preloaded with $\left\{\pi ,ID_{BS},c_{BS},ID_{H_{s}},c_{H_{s}},K_{H_{s}}^{U},ID_{L_{i}},K_{L_{i}}^{R},\left(R_{L_{i}},d_{L_{i}}\right)\right\}$, where $K_{H_{s}}^{U}$ represents the public keys of the ECC encryption algorithm [21] for all H-sensors and $K_{L_{i}}^{R}$ represents the private key of the ECC encryption algorithm for each L-sensor. Each H-sensor will preload $\left\{\pi ,ID_{BS},c_{BS},ID_{H_{i}},\left(R_{H_{i}},d_{H_{i}}\right),K_{H_{i}}^{R},ID_{L_{s}},c_{L_{s}},K_{H}\right\}$, where $K_{H_{i}}^{R}$ is the private key of the ECC encryption algorithm of each H-sensor and $K_{H}$ is used for communication between H-sensors (and BS). The BS is preloaded with $\left\{s,\pi ,{\mathrm{ID}}_{BS},\left(R_{BS},d_{BS}\right),ID_{H_{s}}\;c_{H_{s}},\left(R_{H_{s}},d_{H_{s}}\right),ID_{L_{s}},c_{L_{s}},\left(R_{L_{s}},d_{L_{s}}\right),K_{L_{s}}^{U},K_{H}\right\}$, where $K_{L_{s}}^{U}$ represents the public keys of the ECC encryption algorithm for all L-sensors.

#### 3.2.4. Routing Structure Generation

In HWSNs, the routing structure consists of two parts: Intra-cluster routing and inter-cluster routing. The former means that the intra-cluster L-sensor uploads the collected perceptual data to the nearest CH through the shortest path algorithm. The latter refers to the routing protocol where the CH uploads the collected sensory data to the BS according to the shortest path algorithm. We apply wireless sensor networks to military environmental monitoring. It is assumed that the surveillance area is a square area of 1000 m × 1000 m, and a trusted and protected BS is established in the center of the area in advance. Please note that our scheme does not restrict the BS to the regional center, but the communication range of the BS must cover the area. Then, a military aircraft randomly and uniformly spreads 20 H-sensors and 980 L-sensors in the surveillance area. Each L-sensor (and H-sensor) can obtain its own location through some sort of location service (such as [22,23]). Considering that the energy consumption and cost of GPS are not suitable for WSNs, we do not use the GPS location service.

**Figure 1:**
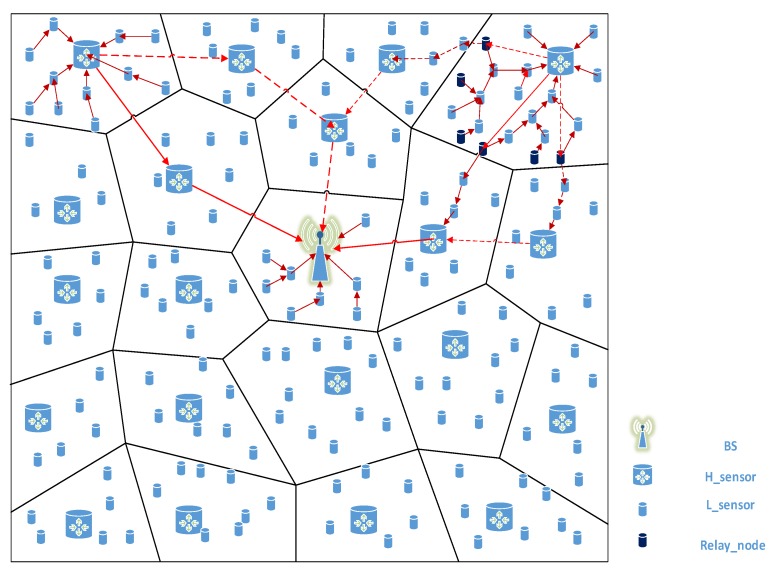
System model.

In our scheme, the routing structure design within the network requires the BS to collect the location information of nodes in the entire network in advance. First, the BS broadcasts the invitation-join message (including ${\mathrm{ID}}_{BS}$ and timestamp $T_{BS}$) to all nodes in the network by means of global broadcast. Then, once the H-sensor receives the invitation-join message, each H-sensor (say $H_{1}$) will locally broadcast a Hello message (including $ID_{H_{1}}$ and timestamp $T_{H_{1}}$). Each L-sensor determines which H-sensor it belongs to by analyzing the signal strength of the received Hello message. (We assume that some L-sensors choose $H_{1}$ as the CH). Take u in these L-sensors as an example. u uploads its join-reply message (including $ID_{u}$, location information $l_{u}$, timestamp $T_{u}$, and digital signature $\sigma_{u}$). What we need to pay attention to is that $l_{u}$ needs to encrypt with $K_{H_{1}}^{U}$. Finally, after $H_{1}$ receives $\sigma_{u}$, it uses the $ID_{u}$ preloaded to verify $\sigma_{u}$. Other nodes perform similar operations. $H_{1}$ encrypts the encrypted join-reply messages of these L-sensors with $K_{H}$ and uploads them to BS. In Figure 1, we show a system model diagram of the entire network routing structure design.

Although the nodes in this area are randomly and evenly distributed, there is no guarantee that some communication nodes must be within their own communication range. Therefore, there are two problems in the process of uploading join-reply messages to the BS in the network: (1) Some special L-sensors may not receive Hello messages broadcast by H-sensors. (2) There may be special H-sensors that cannot communicate directly with all surrounding H-sensors, resulting in an interrupt to upload the join-reply messages.

In order to prevent some L-sensors in the network from becoming isolated nodes because they cannot receive Hello messages from H-sensors, we use the surrounding L-sensors to help these L-sensors complete the join-reply message upload. When there is no direct communication between H-sensors, we select some relay L-sensors between H-sensors to complete the upload of the join-reply messages. The specific solutions to these two problems are as follows:

For the first problem, the BS can notify all L-sensors (including these special L-sensors) through global broadcast. After receiving the invitation-join message, these special L-sensors through local broadcast send their own join-reply messages to the L-sensor that can receive the H-sensor signal strength, and they then use the L-sensor to indirectly upload their own join-reply messages to the H-sensor. For the second problem, these special H-sensors (say $H_{1}$) first decrypt the join-reply messages of these L-sensors with $K_{H_{1}}^{R}$ and then find the appropriate relay nodes by the position information of these L-sensors. The nodes in our scheme are randomly deployed, so CH can only find some relay nodes from the location information of the collected L-sensors. This is different from some schemes (such as [24,25]) using manual deployment of relay nodes to enhance network connectivity. Finally, $H_{1}$ can indirectly upload the collected join-reply messages (encrypted with $K_{H}$) of these L-sensors to the surrounding H-sensors by means of relay nodes. The BS will receive join-reply messages of all L-sensors. After completing the message collection, the BS generates a network routing structure using the location information of all L-sensors in combination with the shortest path algorithm.

**Figure 2:**
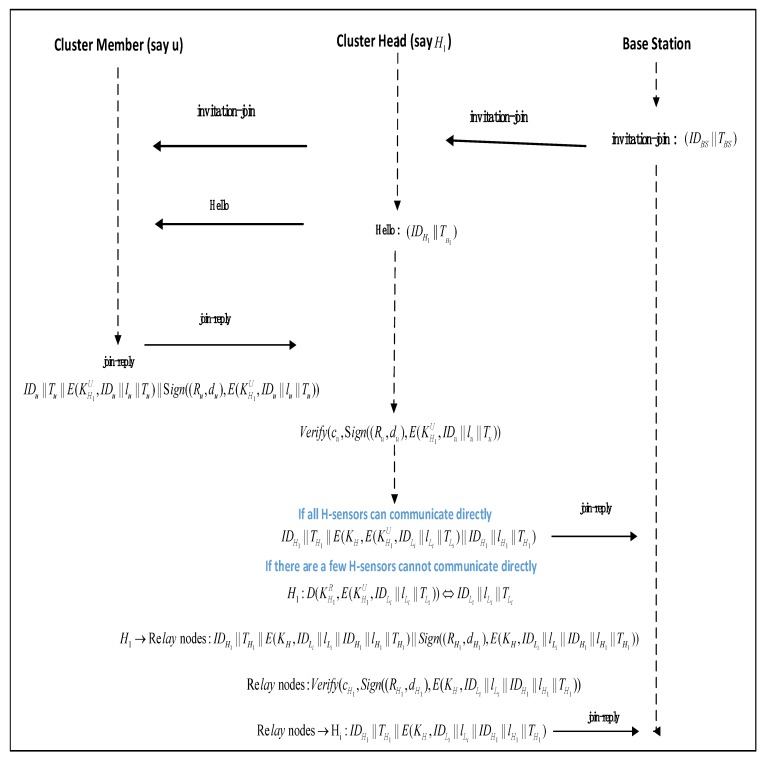
The process of nodes in the network uploading their own location information.

The BS determines the communication neighbor relationship between nodes according to the network routing structure. Next, the BS can help the node establish a session key according to the communication neighbor relationship of the nodes in the network. In Table 1, we describe some security measures identifiers. In Figure 2, we visually demonstrate the process by which the L-sensor in the network uploads its own location information to the BS.

### 3.3. Key Establishment Process

#### 3.3.1. Centralized Session Key Establishment

It is assumed that the L-sensor (say u, v) will be determined to be the communication neighbor node relationship. The BS first generates a session key $K_{uv}$ for u and v through a pseudo-random function. Then, $K_{uv}$ is encrypted by $K_{u}^{U}$ and $K_{v}^{U}$, respectively. Finally, the encrypted $K_{uv}$ is digitally signed with $\left(R_{BS},d_{BS}\right)$ to obtain $\sigma_{BS}$. The BS sends $\sigma_{BS}$ to u and v by unicast. When the two nodes receive $\sigma_{BS}$, they use the preloaded $c_{BS}$ to perform digital signature verification and then use their own $K_{u}^{R}$ and $K_{v}^{R}$ to decrypt and obtain $K_{uv}$, respectively.

#### 3.3.2. Distributed Session Key Establishment 

The assumption of the communication neighbor node relationship is consistent with the above. The BS first encrypts the routing materials of u and v with $K_{u}^{U}$ and $K_{v}^{U}$, respectively. Then, the BS digitally signs the encrypted routing material with $\left(R_{BS},d_{BS}\right)$ to obtain $\sigma_{BS}$. Finally, the BS sends $\sigma_{BS}$ to u and v by unicast, respectively. When the two nodes receive $\sigma_{BS}$, they use the preloaded $c_{BS}$ to perform digital signature verification and then use their own $K_{u}^{R}$ and $K_{v}^{R}$, respectively, to decrypt and obtain their own routing materials.

The routing material of each L-sensor includes the ${\mathrm{ID}}_{i}$ and $c_{L_{i}}$ of its optimal communication neighbor node and the ${\mathrm{ID}}_{j}$ and $c_{L_{j}}$ of the backup communication neighbor node (relay nodes may contain IDs of multiple backup communication neighbor nodes). In the early stage, the optimal neighbor node will be the first object to establish the session key. However, the process of establishing session keys between nodes requires authentication to prevent some attacks (such as [26]). Taking nodes u and v as examples, combining the principle of the PF-IBS algorithm, the key establishment process of $K_{uv}$ requires the following five steps: 

**STEP 1:**u selects $\varphi_{u},r_{u}\in {}_{R}Z_{*}^{q}$, calculates $E_{u}=\varphi_{u}P$ first, and then calculates $h_{u}=H_{2}\left(m_{u},ID_{u},R_{u}\right)$ and $Z_{u}=\varphi_{u}+h_{u}d_{u}\;mod\;q$, where $m_{u}=r_{u}P$. Finally, u sends $\left\langle ID_{u},E_{u},R_{u},Z_{u},m_{u},T_{u}\right\rangle$ to v.

**STEP 2:** After obtaining the message from u, v first determines if $T_{u}$ is valid, and, if it expires, rejects the message. It is determined whether $ID_{u}$ is consistent with the $ID_{u}$ distributed by BS. If the confirmation is consistent, then u is a communication neighbor node. Next, v calculates $h_{u}=H_{2}\left(m_{u},ID_{u},R_{u}\right)$ and determines whether the left and right sides of $Z_{u}P=E_{u}+h_{u}\left(R_{u}+c_{u}P_{pub}\right)$ are equal. If the two sides are equal, $m_{u}$ and identity authentication pass, and vice versa. It should be noted that the BS has assigned the public key $c_{u}$ of u to v.

**STEP 3:**v selects $\varphi_{v},r_{v}\in {}_{R}Z_{q}^{*}\;$, calculates $E_{v}=\varphi_{v}P$ first, and then calculates $h_{v}=H_{2}\left(m_{v},ID_{v},R_{v}\right)$ and $Z_{u}=\varphi_{u}+h_{u}d_{u}\;mod\;q$, where $m_{v}=r_{v}P$. Then, v sends $\left\langle ID_{v},E_{v},R_{v},Z_{v},m_{v},T_{v}\right\rangle$ to u.

**STEP 4:** After obtaining the message from v, u first determines if $T_{v}$ is valid, and, if it expires, rejects the message. It is determined whether ${\mathrm{ID}}_{v}$ is consistent with the ${\mathrm{ID}}_{v}$ distributed by BS. If the confirmation is consistent, then v is a communication neighbor node. Next, u calculates $h_{v}=H_{2}\left(m_{v},ID_{v},R_{v}\right)$ and determines whether the left and right sides of $Z_{v}P=E_{v}+h_{v}\left(R_{v}+c_{v}P_{pub}\right)$ are equal. If the two sides are equal, $m_{v}$ and identity authentication pass, and vice versa.

**STEP 5:**u and v respectively generate a shared key $K_{uv}=r_{u}\cdot r_{v}P=r_{v}\cdot r_{u}P=K_{vu}$.

#### 3.3.3. New Node Key Establishment and Old Key Deletion

In order to achieve the shortest path for the L-sensor to upload data, some L-sensors in the routing structure will be the optimal communication neighbor node of multiple L-sensors. There may be some L-sensors that act as relay nodes for inter-cluster data uploading. These optimal communication neighbor nodes and relay nodes undertake too many data upload tasks. They die prematurely due to excessive energy expenditure. There are even some L-sensors that are captured by the attacker. The death or capture of certain nodes can result in severe network partitioning, which prevents uploading of collected data. In order to extend the network life cycle, we need to revoke them and add new L-sensors. We assume that there is a detection mechanism (such as [27,28]) in the network to screen out death or captured nodes in the network. The BS notifies all L-sensors in the network about the IDs of these nodes by means of global broadcast. All L-sensors will check the IDs of their communication neighbor nodes. If IDs of these nodes are found, the L-sensor will delete IDs of these nodes and the previously established shared keys. When adding some new nodes to a certain area, the BS first encrypts the ${\mathrm{ID}}_{i}$ of the new node with the $c_{L_{j}}$ of the specific node of the area. Then, it uses ($R_{BS}$, $d_{BS}$) to digitally sign the encrypted message to obtain $\sigma_{BS}$. Finally, it sends the $\sigma_{BS}$ to the specific node in the area by unicast, and these specific nodes will obtain IDs of new nodes. The new node (preloading IDs of the specific node) uses the above-mentioned distributed key establishment method to complete key establishment between the specific node and the new node.

#### 3.3.4. Routing Update

In our scheme, the BS periodically informs nodes in the network to upload their remaining energy values. After obtaining the remaining energy values of all nodes, the BS will update the routing structure according to the remaining energy value of the node and the path energy consumption values. Next, according to the new routing table, the BS notifies the L-sensor to update the communication neighbor by unicast and completes the new session keys establishment.

## 4. Performance Evaluation

### 4.1. The Comparison of Key Storage Cost

In [11], Du et al. assume that in HWSNs, the number of H-sensors and L-sensors is M and N, respectively, and satisfies M << N. The following other schemes have the same assumptions on the number of nodes. In this scheme, each L-sensor (such as u) preloads its own private key and CH’s public key. Each H-sensor preloads its own private key, u’s public key, and key $K_{H}$. The number of keys preloaded by Du et al.’s scheme is

(1) N×2+M×3=2N+3M.

In [13], Boujelben et al. adopted a pairing idea of bilinear mapping during the key establishment process to assist the node in completing the session key establishment. This scheme has the smallest key storage space occupation. Each H-sensor and each L-sensor only need to store its own private key preloaded by the key distribution center. The number of keys preloaded by Boujelben et al.’s scheme is

(2) M×1+N×1=M+N.

In [15], according to the design process of Wang et al.’s scheme, each L-sensor only preloads its own private key $d_{L_{i}}\;$. Each H-sensor only preloads its own private key $d_{H_{i}}$ and $K_{H}$. Thus, in the scheme of Wang et al., the number of keys preloaded is

(3) N+M×2=N+2M.

In [17], Harbi et al. designed a layered sensor network key management scheme. As can be seen from the node initialization phase of the scheme, each L-sensor also preloads a master private key k and the BS’s public key ${\mathrm{Pu}}_{BS}$, and each H-sensor preloads a master private key k and the BS’s public key ${\mathrm{Pu}}_{BS}$. In this scheme, the BS is considered a powerful device; therefore, all keys preloaded to the BS are negligible. The number of keys preloaded by Harbi et al.’s layered sensor network key management scheme is

(4) N×2+M×2=2N+2M.

In our scheme, the BS preloads a lot of keys, which play a very important role. It should be emphasized that the BS has enough storage space; thus, all keys preloaded to the BS are negligible. In terms of preloading keys, our centralized key management scheme is the same as the distributed key management scheme. Each L-sensor is preloaded with $\left\{c_{BS},c_{H_{s}},K_{H_{s}}^{U},\left(R_{L_{i}},d_{L_{i}}\right),K_{L_{i}}^{R}\right\}$, and each H-sensor preloads $\left\{c_{BS},\left(R_{H_{i}},d_{H_{i}}\right),K_{H_{i}}^{R},c_{L_{s}},K_{H}\right\}$. In our scheme, the number of keys preloaded is

(5) N×(2×M+3)+M×(4+N)=3MN+3N+4M.

**Figure 3:**
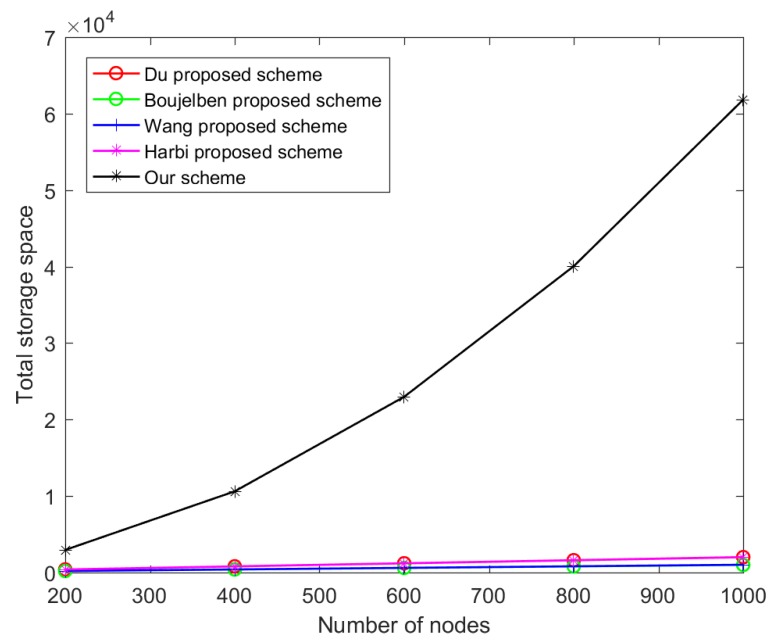
The comparison of key storage cost.

In Table 2, we show the distribution ratio between the H-sensor and the L-sensor in HWSNs. Figure 3 shows the comparison of the above schemes in terms of key preloading.

In Figure 3, the total key storage cost is higher in our scheme, but the storage cost of a single L-sensor can withstand the number of preloaded keys. We assume that the length of a single key is 160 bits. We follow the configuration of the fifth set of nodes in Table 2. A single L-sensor needs 0.86 KB to store the preloaded keys and a single H-sensor needs 19.68 KB to store the preloaded keys. We know that MICA2 has 128 KB of storage space, so the L-sensor can withstand the number of preloaded keys. The storage space of a single H-sensor itself is larger than the storage space of a single L-sensor, so the H-sensor can withstand the number of preloaded keys. Our scheme occupies more total key storage space for several reasons: (1) In our scheme, each L-sensor does not know which H-sensor will manage it before deployment. Therefore, it is essential that each L-sensor preloads $K_{H_{s}}^{U}$ in advance. $K_{H_{s}}^{U}$ and $K_{H_{i}}^{R}$ can be used to encrypt the location information of the protected node to prevent attackers from eavesdropping on the node’s private information. $K_{L_{i}}^{R}$ will be used to protect the session key or routing material of the L-sensor assigned by the BS. (2) In Wang’s scheme, the scheme assumes that the L-sensor and the H-sensor in the cluster know the neighbors in advance. This indicates that the scheme is applicable to a network model in which the node knows the deployment knowledge rather than the randomly deployed network model, because randomly deployed nodes cannot know in advance which nodes are communication neighbors [29]. In order to obtain the deployment knowledge of the network model, our scheme needs to store $c_{BS}$ to complete the digital signature authentication to obtain the routing structure from the BS. (3) We do not use the IBE algorithm to protect the L-sensor location information. Although this approach eliminates the need to preload $K_{H_{s}}^{U}$ and $K_{H_{i}}^{R}$, the use of bilinear mapping operation in the IBE algorithm consumes more energy than the ECC encryption algorithm. Due to space limitations, we can refer to some papers [30,31] to understand the IBE algorithm.

### 4.2. The Comparison of Computation Cost

**Figure 4:**
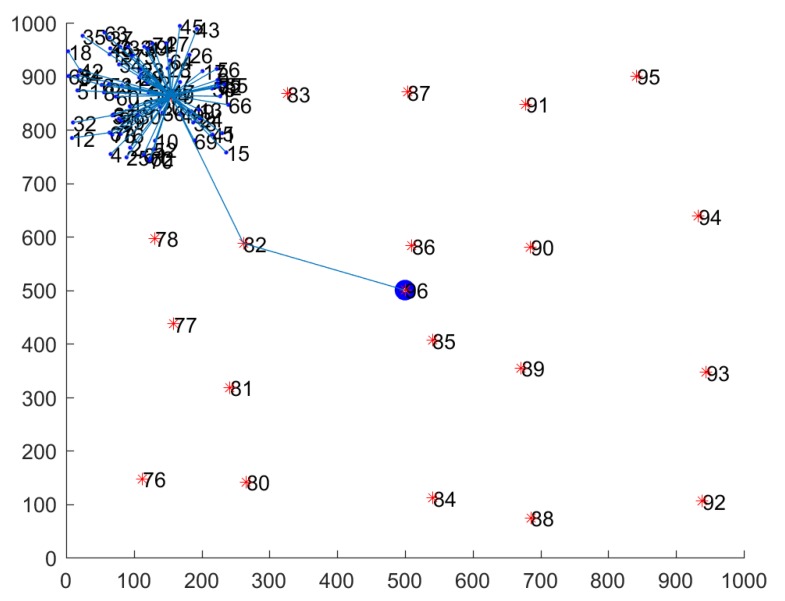
Network routing structure simulation diagram.

According to the HWSNs model we designed, we use MATLAB to simulate a cluster-generated routing structure to study. As the distribution of nodes in a single cluster is the same as the distribution of nodes in the entire network, in order to obtain the computing cost of the network model faster, we take a single cluster as the research object. Figure 4 shows a routing structure diagram of nodes within a cluster in the case of a single cluster. In Figure 4, the cluster contains 1 H-sensor and 75 L-sensors.

**Figure 5:**
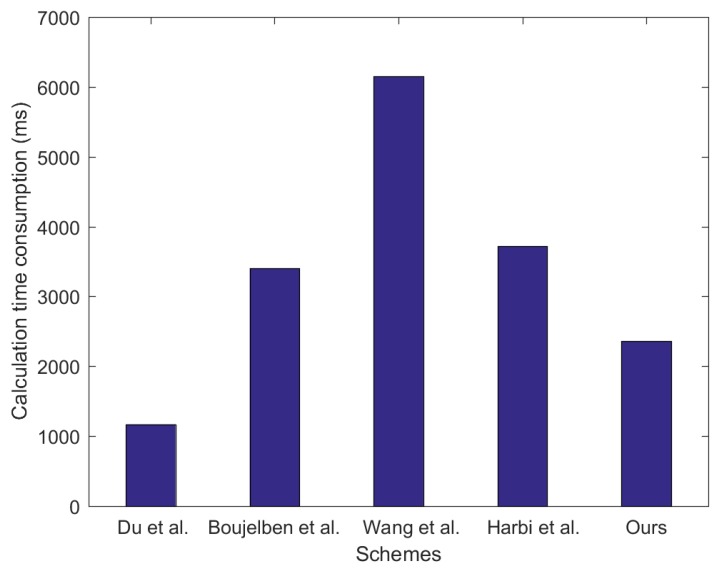
The comparison of calculation costs of the scheme.

To compare the computational costs, we use the paper [32] referenced by Harbi et al. to obtain some important calculation parameters. The acquisition of important calculation parameters in [32] is derived from the PBC library based on the GMP library. We adopt the same energy consumption comparison idea as the scheme [11,12,15], that is, we only consider the calculation cost of the key without considering the cost of data communication. Table 3 shows the time required for various calculation operations in the designed algorithm. Figure 5 shows the comparison of the energy consumed by our scheme with other schemes during the key establishment process.

In Figure 5, our scheme has a lower computational cost than schemes [13,15,17]. Du et al.’s scheme lacks location information protection and message authentication during the session key establishment process and, thus, has the lowest computational cost. However, its security is very poor. In order to improve the security of the established session key, the security authentication mechanism is very important, such as [33]. Our scheme has lower calculation costs for several reasons: (1) In our scheme, the public key of the communication neighbor node is pre-allocated by the BS, thereby saving the computational cost of the node performing the hash function operation on G. (2) Our scheme uses the PF-IBS algorithm, which saves the computational cost of bilinear pairing operations.

### 4.3. Security Performance Discussion

- (1) Forward secrecy of master private key. Forward secrecy: The private key of one or more participating entities is compromised, but the established session key is not destroyed. In Harbi et al.’s scheme, once s is leaked, all session keys will be compromised, so the scheme does not have the forward secrecy of the master private key. However, in our scheme, even if s is leaked, it does not affect the shared key that has been established.
- (2) Resist replay attacks. Replay attacks: The attacker misleads the legitimate node by resending the previous authentication code and synchronizing it to the wrong time. In our scheme, the message forwarded by the node adds a timestamp, which ensures the freshness of the data and prevents the attacker from initiating replay attacks.
- (3) Resist the node replication attack. Replication attack: The attacker captures the node and places a copy of it in multiple geographic locations to establish the illegal communication link with the legitimate node. There are some schemes (such as [34,35]) for preventing the node replication attacks. In our scheme, the BS pre-allocates information about the communication neighbor nodes of each node within the network. At the same time, we adopt the neighbor node authentication mechanism, and the legal node refuses to receive the information of the replica node, so it cannot pass the authentication and establish a secure communication link. Therefore, our scheme can effectively resist node replication attacks.
- (4) Resisting node capture attack. Resilience: Probability of exposing keys of the uncaptured node when some nodes are captured. The lower the resilience value, the more difficult it is for an attacker to exploit the useful information of the captured node to attack legitimate nodes. Conversely, the more nodes the attacker captures, the more useful information will be obtained and the higher the resilience value. In our scheme, the attacker cannot obtain the key of the uncaptured node by the information of the captured node.
- (5) Network weak area protection: Protection of relay node location information. Similar to the need to protect some important private information in our lives (such as [36,37]), we need to protect some important data information from being leaked. However, the location information protection of the node in our scheme is different from the privacy protection of the source nodes mentioned in the paper [38]. We know that the source node location privacy protection scheme for homogeneous WSNs has achieved some research results, but the source node location privacy protection scheme for heterogeneous wireless sensor networks has not been studied. However, the protection of the source node location privacy is not the focus of our scheme. Our scheme focuses on encrypting the location information uploaded by the L-sensor to prevent an attacker from obtaining a global routing table for the network. When an attacker obtains a global routing table, it is easy to find the location of the relay node. The number of these relay nodes is very limited. As the number of captured relay nodes increases, it will seriously affect the data upload in the network, and even lead to network partitioning. Our scheme prevents attackers from eavesdropping on the location information of the node of the network to generate a global routing table to find relay nodes. Table 4 shows the discussion of various schemes about security attacks.

Table 4 discusses the security performance of the various schemes. In order to ensure that the scheme is more secure, it is indispensable for the proposed scheme to incorporate a secure authentication process. Although the identity-based security mechanism has many advantages in the message authentication process, it is essential to ensure the security of the master private key. At the same time, when we make full use of the characteristics of the network model to design the scheme, we must pay attention to protecting the privacy information of the network nodes.

## 5. Conclusions 

In this paper, we presented a key management scheme based on the PF-IBS algorithm for HWSNs. In our scheme, the BS acted as a data processing center to accomplish the tasks of routing structure generation, routing material allocation, and routing updates, thereby saving a lot of computational cost for the internal network. We used the PF-IBS algorithm to perform authentication. As the algorithm does not require bilinear pairing operations, it can save a lot of computational cost compared to other authentication schemes with bilinear pairing operations. Our scheme reasonably sacrificed some storage space, but ensured network security and saved energy. In the future, we will examine some of the problems faced by key management under new routing protocols and mobile node network models. 

## Figures and Tables

**Table 1:** Notation for security protocols.

Notation	Description
E(k,m)	Asymmetric encryption of m using the key k
D(K,m)	Asymmetric decryption of m using the key K
Sign(k,m)	Digital signature of m using the key k
Verify(K,m)	Signature verification of m using the key K

**Table 2:** Proportion of two types of nodes in heterogeneous wireless sensor networks (HWSNs).

Sensor Type:	1	2	3	4	5
H-sensor	4	8	12	16	20
L-sensor	196	392	588	784	980

**Table 3:** Various calculation operation times in the algorithm.

Operation Style	Time (ms)
Point addition calculation on ECC	0.0288
Scalar multiplication calculation on ECC	2.226
Hash function calculation on G	12.419
Bilinear pairing calculation on G	5.811
One-way hash function calculation	0.0023
Encryption calculation-based ECC	4.452
Decryption calculation-based ECC	2.226
Encryption/decryption calculation-based IBE	3.85
Symmetric encryption/decryption calculation	0.0046

**Table 4:** Discussion of various schemes about security performance.

Various Schemes	Du et al. [11]	Boujelben et al. [13]	Wang et al. [15]	Harbi et al. [17]	Ours
Forward secrecy	Yes	Yes	Yes	No	Yes
Replay attack	Yes	No	Yes	Yes	No
Replication attack	Yes	No	No	No	No
Capture attack	No	No	No	Yes	No
Relay node attack	Yes	Yes	Yes	Yes	No

## References

[1] Kandris D., Nakas C., Vomvas D., Koulouras G. (2020). Applications of Wireless Sensor Networks: An Up-to-Date Survey. Appl. Syst. Innov..

[2] Yang Y., Liu X., Deng R.H., Li Y. (2020). Lightweight Sharable and Traceable Secure Mobile Health System. IEEE Trans. Dependable Secur. Comput..

[3] Cheng S., Wang L., Ao N., Han Q. (2020). A Selective Video Encryption Scheme Based on Coding Characteristics. Symmetry.

[4] Gura N., Patel A., Wander A., Eberle H., Shantz S.C. Comparing elliptic curve cryptography and RSA on 8-bit CPUs. Proceedings of the International Workshop on Cryptographic Hardware and Embedded Systems.

[5] Szczechowiak P., Oliveira L.B., Scott M., Collier M., Dahab R. NanoECC: Testing the limits of elliptic curve cryptography in sensor networks. Proceedings of the European Conference on Wireless Sensor Networks.

[6] Shamir A. Identity-Based Cryptosystems and Signature Schemes. Proceedings of the Advances in Cryptology.

[7] Traynor P., Kumar R., Choi H., Cao G., Zhu S., Porta T.L. (2007). Efficient Hybrid Security Mechanisms for Heterogeneous Sensor Networks. IEEE Trans. Mob. Comput..

[8] Alagheband M.R., Aref M.R. (2012). Dynamic and secure key management model for hierarchical heterogeneous sensor networks. IET Inf. Secur..

[9] Mahmood Z., Ning H., Ghafoor A. (2017). A polynomial subset-based efficient multi-party key management system for lightweight device networks. Sensors.

[10] Rezapour T.Y., Ebrahimi A.R., Abolghasemi M.S. (2016). A novel key management scheme for heterogeneous sensor networks based on the position of nodes. Isecure Isc Int. J. Inf. Secur..

[11] Du X., Guizani M., Xiao Y., Chen H.H. (2009). Transactions papers a routing-driven elliptic curve cryptography based key management scheme for heterogeneous sensor networks. IEEE Trans. Wirel. Commun..

[12] Du X., Xiao Y., Ci S., Guizani M., Chen H.H. A Routing-Driven Key Management Scheme for Heterogeneous Sensor Networks. Proceedings of the IEEE International Conference on Communications, ICC 2007.

[13] Boujelben M., Youssef H., Mzid R., Abid M. (2011). IKM—An Identity based Key Management Scheme for Heterogeneous Sensor Networks. J. Commun..

[14] Nan L. Research on Diffie-Hellman key exchange protocol. Proceedings of the 2010 2nd International Conference on Computer Engineering and Technology.

[15] Wang J.R., Wang H.F. Distributed Key Management Scheme Based on ECC for Heterogeneous Sensor Networks. Proceedings of the 2014 Second International Conference on Advanced Cloud and Big Data (CBD).

[16] Sharma D., Bhondekar A.P. (2018). Traffic and Energy Aware Routing for Heterogeneous Wireless Sensor Networks. IEEE Commun. Lett..

[17] Harbi Y., Aliouat Z., Refoufi A. (2019). Enhanced Authentication and Key Management Scheme for Securing Data Transmission in the Internet of Things. Ad Hoc Netw..

[18] Mehmood A., Umar M.M., Song H. (2017). ICMDS: Secure inter-cluster multiple-key distribution scheme for wireless sensor networks. Ad Hoc Netw..

[19] Sharma G., Bala S., Verma A.K. (2017). PF-IBS: Pairing-free identity based digital signature algorithm for wireless sensor networks. Wirel. Pers. Commun..

[20] Zhu S., Setia S., Jajodia S. (2006). LEAP+: Efficient security mechanisms for large-scale distributed sensor networks. ACM Trans. Sens. Netw..

[21] Almajed H.N., Almogren A.S. (2019). SE-Enc: A Secure and Efficient Encoding Scheme Using Elliptic Curve Cryptography. IEEE Access.

[22] Manickam M., Selvaraj S. (2019). Range-based localisation of a wireless sensor network using Jaya algorithm. IET Sci. Meas. Technol..

[23] Mao G., Fidan B., Anderson B.D. (2007). Wireless sensor network localization techniques. Comput. Netw..

[24] Djenouri D., Bagaa M. (2017). Energy-aware constrained relay node deployment for sustainable wireless sensor networks. IEEE Trans. Sustain. Comput..

[25] Wang F., Wang D., Liu J. (2011). Traffic-Aware Relay Node Deployment: Maximizing Lifetime for Data Collection Wireless Sensor Networks. IEEE Trans. Parallel Distrib. Syst..

[26] Wang M.W., Wang L.J., Yang Q.H., Xie W.M. (2014). Realizing a Mutual Authentication Scheme Base on Telosb in Wireless Sensor Networks. J. Softw. Eng..

[27] Fan L., Wang L. (2014). Intrusion Detection System Based on Integration of Neural Network for Wireless Sensor Network. J. Softw. Eng..

[28] Zidi S., Moulahi T., Alaya B. (2018). Fault Detection in Wireless Sensor Networks Through SVM Classifier. IEEE Sens. J..

[29] Xiao Y., Rayi V.K., Sun B., Du X., Hu F., Galloway M. (2007). A survey of key management schemes in wireless sensor networks. Comput. Commun..

[30] Guo F., Mu Y., Susilo W., Hsing H., Wong D.S., Varadharajan V. (2015). Optimized identity-based encryption from bilinear pairing for lightweight devices. IEEE Trans. Dependable Secur. Comput..

[31] Libert B., Quisquater J.J. On Constructing Certificateless Cryptosystems from Identity Based Encryption. Proceedings of the 9th International Conference on Theory and Practice of Public-Key Cryptography.

[32] Kilinc H.H., Yanik T. (2013). A survey of SIP authentication and key agreement schemes. IEEE Commun. Surv. Tutor..

[33] Wazid M., Das A.K., Bhat V., Vasilakos A.V. (2020). LAM-CIoT: Lightweight authentication mechanism in cloud-based IoT environment. J. Netw. Comput. Appl..

[34] Xie W., Wang L., Wang M. (2014). A Bloom Filter and Matrix-based Protocol for Detecting Node Replication Attack. J. Netw..

[35] Li L., Xu G., Jiao L., Li X., Wang H., Hu J., Xian H., Lian W., Gao H. (2020). A Secure Random Key Distribution Scheme Against Node Replication Attacks in Industrial Wireless Sensor Systems. IEEE Trans. Ind. Inform..

[36] Du A., Wang L., Cheng S., Ao N. (2020). A Privacy-Protected Image Retrieval Scheme for Fast and Secure Image Search. Symmetry.

[37] Li C., Zhang Y., Xie E.Y. (2019). When an attacker meets a cipher-image in 2018: A year in review. J. Inf. Secur. Appl..

[38] Jiang J., Han G., Wang H., Guizani M. (2019). A survey on location privacy protection in Wireless Sensor Networks. J. Netw. Comput. Appl..

